# Comparison of Different Drying Methods for Recovery of Mushroom DNA

**DOI:** 10.1038/s41598-017-03570-7

**Published:** 2017-06-07

**Authors:** Shouxian Wang, Yu Liu, Jianping Xu

**Affiliations:** 1 Department of Biology, McMaster University, Hamilton, ON L8S 4K1 Canada; 2 Institute of Plant and Environment Protection, Beijing Academy of Agriculture and Forestry Sciences, and Beijing Engineering Research Center for Edible Mushrooms, Beijing, 100097 China

## Abstract

Several methods have been reported for drying mushroom specimens for population genetic, taxonomic, and phylogenetic studies. However, most methods have not been directly compared for their effectiveness in preserving mushroom DNA. In this study, we compared silica gel drying at ambient temperature and oven drying at seven different temperatures. Two mushroom species representing two types of fruiting bodies were examined: the fleshy button mushroom *Agaricus bisporus* and the leathery shelf fungus *Trametes versicolor*. For each species dried with the eight methods, we assessed the mushroom water loss rate, the quality and quantity of extracted DNA, and the effectiveness of using the extracted DNA as a template for PCR amplification of two DNA fragments (ITS and a single copy gene). Dried specimens from all tested methods yielded sufficient DNA for PCR amplification of the two genes in both species. However, differences among the methods for the two species were found in: (i) the time required by different drying methods for the fresh mushroom tissue to reach a stable weight; and (ii) the relative quality and quantity of the extracted genomic DNA. Among these methods, oven drying at 70 °C for 3–4 h seemed the most efficient for preserving field mushroom samples for subsequent molecular work.

## Introduction

Specimens in herbaria are a valuable source of materials not only for morphological studies, but also for molecular ecology, population genetics, population genomics, and systematics investigations^1–6^. For research requiring genetic materials from herbarium specimens, the method by which specimens are dried can have a significant impact on the quality and quantity of DNA extracted. For oven-dried specimens, two factors, drying temperature and length of drying time, could impact DNA quantity and quality^2, 7–9^. However, studies have primarily focused on plant materials and there is little information about the effectiveness of different drying methods for mushrooms. Identifying effective methods for drying wild mushrooms is important because for many mushroom species, there is currently no method for growing their pure mycelial cultures for molecular investigations. As a result, their genetic and genomic studies rely almost exclusively on dried fruiting body specimens from natural environments. To preserve plant and mushroom specimens, drying using silica gel has become common among field biologists, especially in remote areas where electricity may be scarce^10, 11^. However, at present, the effectiveness of silica gel in drying plant leaves is controversial. For example, Liston *et al*.^12^ reported that fresh air-dried spinach (*Spinacia oleracea*) leaves stored at 37 °C for over four months showed less DNA degradation than fresh spinach placed directly in silica gel and stored in the silica gel at 37 °C for four months. In contrast, Harris^13^ reported no difference between oven-drying at 70 °C and drying with silica gel at room temperature for leaf samples from *Vochysiaceae*, *Coffea*, and *Leguminosae* plants. However, such comparisons are not available for mushroom specimens.

Mycologists have traditionally followed protocols used by plant biologists for drying mushroom (macrofungi) specimens. However, mushroom specimens differ from plant specimens in many aspects. For example, the water content of fresh fleshy mushrooms is typically much higher than that in fresh leaves in plants. During high-temperature drying, the high water content and vapor in mushrooms might accelerate the degradation of DNA. To eliminate the water vapor effect, Hosensey^14^ first introduced the silica gel method for drying mushrooms. Haines and Cooper^15^ also assessed the amounts of *Agaricus brunnescens* (syn. *Agaricus bisporus*) DNA after drying at 22 °C, 38 °C and 93 °C, and compared them with several liquid preservation methods (95% ethanol, 5% phenol, 1% HgCl_2_, or 10% formaldehyde). Their results showed that 22 °C was better than 38 °C and that no DNA was found after drying at 93 °C, with the liquid preservation methods being worse than drying at 22 °C and 38 °C but better than drying at 93 °C^15^. Hosaka and Uno^5^ compared the effectiveness of three different oven drying temperatures (35 °C, 52 °C, and 71 °C) for seven mushroom species and concluded that DNA extracted from specimens dried at the three temperatures did not differ significantly in its suitability for subsequent studies. Unfortunately, none of the above studies directly compared the difference between silica gel drying and oven drying at different temperatures and the range of temperatures used for oven drying was relatively limited.

Several methods have been used to assess DNA quality and quantity, including agarose gel electrophoresis^5, 7, 12^, gene-specific PCR^16^, multiplex PCR^17^, RAPD-PCR^18^, Southern hybridization^19^, UV spectrophotometry^20^, and more recently micro-capillary electrophoresis using the Agilent Bioanalyzer^6, 21^, NanoDrop Spectrophotometer and Fluorometer^6, 21–23^. Each of the above-mentioned methods has advantages and disadvantages in terms of specificity, sensitivity, and reproducibility as well as labor, speed and cost for DNA qualification and quantification.

In this study, we dried mushroom samples using two approaches: silica gel drying at ambient temperature and oven drying at a range of temperatures. We then compared the quality and quantity of genomic DNA extracted from these dried specimens. Genomic DNA was extracted from each of the dried specimens at two time points: the first extraction was performed immediately after drying was completed and the second extraction was conducted after the dried samples had been stored at room temperature in a sealed plastic bag for one month. The objective was to investigate the differences among the drying methods in the quality and quantity of genomic DNA from the dried mushroom specimens and to identify the most appropriate/efficient method(s) for drying mushroom samples for DNA-based studies.

## Materials and Methods

### Samples

**Figure 1:**
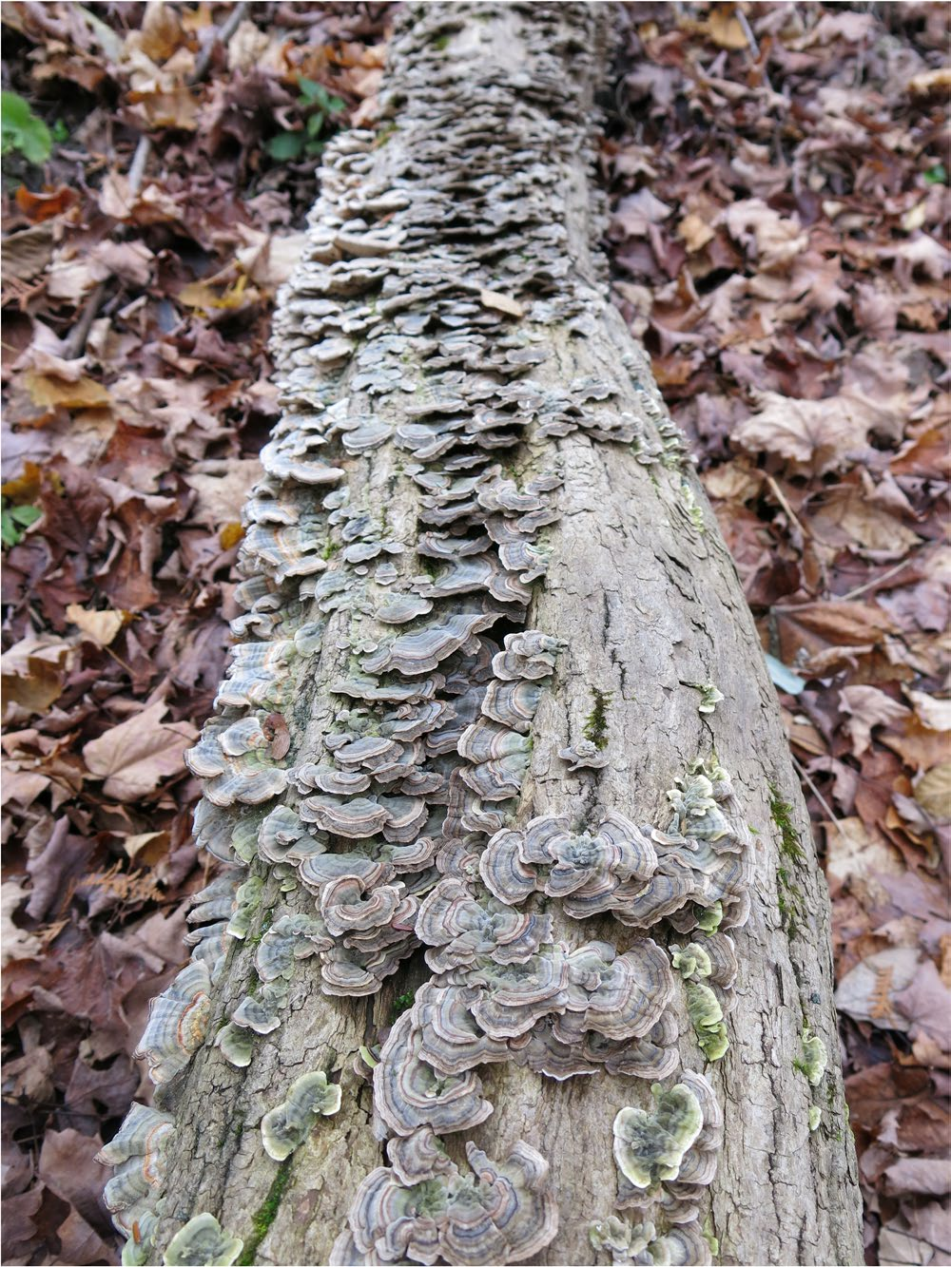
The fruiting bodies of *Trametes versicolor* analyzed in our study.

Two species of mushrooms were investigated in this study: the commercial white button mushroom *Agaricus bisporus* and the shelf fungus *Trametes versicolor*. Because uniform button-stage fruiting bodies are easily obtained for *A*. *bisporus*, this species was chosen as a representative of fleshy mushrooms. For this study, fresh button-stage fruiting bodies of *A*. *bisporus* were obtained from a single grower from a single local supermarket (the Fortinos Supermarket in Hamilton, Ontario, Canada). Similarly, *T*. *versicolor* is a common mushroom in temperate forests. It was chosen as a representative of shelf and leathery mushrooms. In this study, *T*. *versicolor* fruiting bodies were collected from a fallen oak log in a local forest close to McMaster University in Hamilton, Ontario, Canada. There were hundreds of fruiting bodies of *T*. *versicolor* on the log, with most appearing to be at a similar development stage, i.e. mature fruiting bodies (Fig. 1). Throughout our experiment, the log was left in its natural outdoor state in the forest. The weather during our time of study (two weeks) was typical of mid-autumn: cool and without significant prolonged precipitation. Once purchased (for *A*. *bisporus*) or harvested (for *T*. *versicolor*), the fruiting bodies were immediately cut into pieces sized 1 cubic centimeter and 1 square centimeter, respectively. Each piece was separately wrapped in a clean piece of tissue paper (Kimwipes, Kimtech Science, Mississauga, Ontario, Canada) before being dried under the conditions described below.

### Mushroom Drying

For silica gel drying, each wrapped piece of mushroom tissue was separately placed in a sealed Ziploc bag containing dehydrated silica gel pellets (~10 grams in each bag, more than sufficient to absorb 1 ml of water) at 22 °C. For the non-silica gel based drying, a maximum of two ovens were used at any given time, with each set of samples at a different temperature (±0.3 degrees) and the oven temperatures calibrated beforehand for drying the mushroom samples. The wrapped mushroom samples were placed on metal trays in the ovens at 22 °C, 38 °C, 50 °C, 60 °C, 70 °C, 80 °C, and 93 °C, respectively. At specific times after the start of drying (3 h, 6 h, and every four hours after that until a constant weight was reached), the samples were weighed and the water loss ratios were calculated. Here, the water loss ratio (%) was calculated as 100 × (Weight of fresh mushroom − Weight of drying mushroom)/Weight of fresh mushroom.

For each of the drying methods for each species, drying was stopped when the sample reached a constant weight. Both the weight and the approximate drying time (ADT) were recorded for each specimen. After ADTs were obtained for the two species using the different methods, a second set of experiments was carried out to identify a more precise drying time than the ADT. Here, the freshly cut samples prepared and dried as described above were measured at five different time points: (ADT-4) h, (ADT-3) h, (ADT-2) h, (ADT-1) h, and ADT for each of the above drying methods. If the ADT was three hours or less for a tested species-method combination, then the first two drying time points (ADT-4 and ADT-3) were not considered in this second round. After complete drying, a portion of each mushroom tissue piece (40 mg) was immediately processed for nucleic acid extraction while the remaining portion was kept in a sealed plastic bag at room temperature (22 °C) for 1 month before another nucleic acid extraction was conducted. Three replicates were done for each combination of mushroom species-drying method with a total of 48 pieces of mushroom tissue (2 species × 8 treatments × 3 repeats) processed for subsequent DNA quality and quantity studies.

### Nucleic Acid Extractions

Total genomic nucleic acids from each sample were extracted with a modified version of the CTAB method^24^. Briefly, ~40 mg of dried mushroom sample from each piece of dried tissue was weighed and placed into a 2 ml micro-centrifuge tube containing a small number of glass beads (Ø 0.5 mm). The micro-centrifuge tubes were submerged in liquid nitrogen, and the mushroom samples were then ground with sterilized pestles to fine powder. One milliliter of preheated CTAB isolation buffer (2% CTAB, 1.4 M NaCl, 0.2% β-mercaptoethanol, 20 mM EDTA, 100 mM Tris-HCl, pH 8.0) was added to each tube and then incubated at 65 °C for 60 min with occasional gentle mixing. Then, 900 μl of 24:1 chloroform:isoamyl alcohol was added to each tube and mixed gently but thoroughly, followed by centrifugation at 13,000 rpm for 10 min. After centrifugation, 900 μl supernatant was transferred to a new tube, and the chloroform:isoamyl alcohol extraction step was repeated. About 800 µl supernatant was then transferred to a 1.5 ml tube and nucleic acids were precipitated using 500 μl of isopropanol at −20 °C for 24 h. After precipitation, the tubes were spun at 13,000 rpm for 2 min, the supernatant was discarded, and the pellets were washed with 70% ethanol, vacuum dried, and re-suspended in 60 μl 1 × TE buffer. The nucleic acid samples were stored in a −20 °C freezer for subsequent assessment of DNA quality and quantity. A total of 96 DNA extractions were performed in this study, with two extractions separated by one month for each of the 48 dried mushroom samples.

### DNA Quality and Quantity Assessments

The quality and quantity of extracted mushroom DNA samples were assessed using several methods. In the first, the extracted genome nucleic acid samples were visualized by running 5 μl of each extraction on 0.8% agarose gels using Bio-Rad electrophoresis apparatus (Hercules, CA, USA). The gels were then stained with ethidium bromide and visualized under UV using the Alpha-Imager System (Alpha Innovtech Corporation, Hercules, CA, USA). Here, the sizes of the largest and brightest major DNA bands were compared with the standard 1 kb ladder DNA and their relative concentrations were assessed using the spot densitometry function of the Alpha-Imager System.

The second method was UV absorbance based on the spectrophotometer NanoDrop 1000 Instrument (Thermo Fisher Scientific, Wilmington, DE, USA). Both the 260 nm/230 nm and the 260 nm/280 nm ratios were obtained for each of the 96 DNA samples. Pure nucleic acid solutions generally have 260/230 values in the range of 2.0–2.2. If the ratio is lower than that, it may indicate the presence of contaminants such as EDTA, carbohydrates, and phenolic compounds, all of which absorb at 230 nm. For the 260/280 ratio, a value of ~1.8 is generally accepted as “pure” for DNA and a ratio of ~2.0 is generally accepted as “pure” for RNA. A 260/280 ratio lower than 1.8 indicates significant protein contamination.

The third method used the Qubit Fluorometer system for quantification of double-stranded DNA (dsDNA). Because high-temperature drying could have denatured dsDNA in the sample into single strands and the extended low temperature drying environments could have led to hydrolysis and degradation of dsDNA into nucleotides, the readings from NanoDrop 1000 and Qubit Fluorometer were compared to provide an assessment of both the quality and quantity of nucleic acids using the different drying methods. As described above, each treatment had three replicates, and the nucleic acid yield per unit of dried mushroom sample for each replicate was determined.

### Adequacy of Extracted DNA as Template for PCR Amplification

Since PCR amplification is a common method to access extracted mushroom DNA for downstream genotyping and sequencing, we assessed whether there was a difference among nucleic acid samples extracted from mushroom tissues dried by different methods for PCR amplification. Here, we examined the success rate of amplification for two gene fragments, the multi-copy internal transcribed spacer (ITS) of the nuclear ribosomal RNA gene cluster and a single copy nuclear gene.

For PCR amplification of the ITS locus, the primer pairs ITS4 and ITS5 were used and the primer sequences (Table 1) were those described by White *et al*.^25^. The PCR program for amplifying the ITS region was as follows: initial denaturation at 95 °C for 4 min, followed by 35 cycles at 94 °C for 1 min, 55 °C for 1 min and 72 °C for 1 min, and a final extension at 72 °C for 10 min. The template nucleic acid concentration was adjusted to 10 ng/ml. PCR products were sequenced by the McMaster Institute for Molecular Biology and Biotechnology (MOBIX), Canada, to ensure clear chromatographs with no contamination.

**Table 1:** Primers used to assess the adequacy of nucleic acid samples from dried mushrooms as templates for PCR amplification.

Primer	5′-sequence-3′	Source
ITS4	TCCTCCGCTTATTGATATGC	White *et al*.^18^
ITS5	GGAAGTAAAAGTCGTAACAAGG	White *et al*.^18^
AB-F	CCTTCACTCCCATCCCAAGT	This study
AB-R	TGAGGATTGCTCGGGTTCTT	This study
TV-F	ATCATGACGACACGCCAATG	This study
TV-R	GGATCGGTAGTCTGCACGTA	This study

The species-specific single copy gene marker was designed by selecting a region from the largest scaffolds of the respective published genomes of both species (*A*. *bisporus*: estExt_Genewise1.C_100822; *T*. *versicolor*: estExt_fgenesh1_pm.C_7_t10486; http://genome.jgi-psf.org/programs/fungi/index.jsf). The selected genes are annotated as coding for hypothetical proteins and were confirmed as single-copy genes within the *A*. *bisporus* (locus_tag: AGABI2DRAFT_226927) and *T*. *versicolor* (locus_tag: TRAVEDRAFT_59360) genomes using nucleotide BLAST against the respective genome sequences. Primers (for *A*. *bisporus*: AB-F, AB-R; and for *T*. *versicolor*: TV-F, TV-R) were designed for each marker using the online software Primer 3 and the four primers were synthesized by MOBIX at McMaster University. The primer sequences are shown in Table 1. The PCR program for amplifying the single-copy genes was as follows: initial denaturation at 95 °C for 4 min, followed by 40 cycles at 95 °C for 45 s, 50 °C for 45 s and 72 °C for 1 min, and a final extension at 72 °C for 10 min. The template nucleic acid concentrations were adjusted to 10 ng/ml. Sequencing was done by MOBIX to confirm their species identities.

### Statistical Analyses

The drying times for *A*. *bisporus* and *T*. *versicolor* samples for all drying methods were obtained by analyzing the water loss patterns at specific time points until the weights of mushroom tissues had stabilized. The pairwise differences in the quantity and relative quality of nucleic acids extracted from dried specimens were compared using the one-way analysis of variance. Differences among the means of the eight drying methods were assessed using Duncan’s multiple range tests at the 95% confidence level. Statistical analyses were conducted using the evaluation version of SPSS 20.0 for Windows.

Aside from the simple comparisons in DNA quality and quantity among the mushroom drying methods, we also wished to assess the overall effectiveness of the eight methods. At present, there is no consensus on how to assess the overall effectiveness of mushroom sample (or any other biological materials) preparation methods on the quality of nucleic acid extractions. Here, we used a simple approach that took into account how differences in drying time by the various methods affected the quantity of dsDNA over the one-month storage period. The following three equations were used to obtain the indicators of mushroom drying efficacy.

Efficacy of drying based on dsDNA quantity after a specific drying time (EAT) = dsDNA quantity/drying time (in hours).

Relative change of dsDNA quantity over one month storage (RCDQ) = (quantity of dsDNA extracted immediately after drying − quantity of dsDNA extracted one month after drying)/quantity of dsDNA extracted immediately after drying × 100%.

Efficacy of drying based on the relative change of dsDNA quantity over one month storage = RCDQ/drying time (in hours).

## Results and Discussion

### Mushroom Drying Times

In this study, the weights of all mushroom samples had stabilized within 48 h of drying. Tables 2 and 3 show the patterns of water loss ratio over time for *A*. *bisporus* and *T*. *versicolor* fruiting bodies respectively using different drying methods. The bold values represent the final, constant water loss ratios. As expected, the fleshy mushroom *A*. *bisporus* lost more water than the shelf fungus *T*. *versicolor*. Our results showed that the percentages of water loss during drying for *A*. *bisporus* were very similar among the drying methods, ranging from 91.4 to 93.18% (Table 2). In contrast, the percentages of water loss for *T*. *versicolor* showed a broad range among the methods, from 11.92% to 25.01%. For *T*. *versicolor*, drying at the highest tested temperature, 93 °C, led to the greatest water loss while oven drying at 22 °C had the lowest (Table 3).

**Table 2:** The percentage of weight reduction due to water loss during drying of *Agaricus bisporus* fruiting bodies by different drying methods and at different drying times* (mean ± SD, n = 3).

Drying method	Drying time (h)											
	3	6	10	14	18	22	26	30	34	38	42	46
Silica gel at 22 °C	38.08 ± 3.16	53.36 ± 4.11	65.44 ± 4.14	75.30 ± 3.84	82.93 ± 3.53	86.69 ± 2.32	89.65 ± 1.68	91.34 ± 1.16	92.18 ± 0.81	**92**.**86 ± 0**.**60**	92.85 ± 0.60	
Oven at 22 °C	36.25 ± 1.21	52.38 ± 2.00	64.93 ± 2.16	74.66 ± 1.83	81.82 ± 1.50	86.58 ± 1.57	90.03 ± 1.01	91.87 ± 0.26	92.42 ± 0.42	92.63 ± 0.48	**92**.**71 ± 0**.**55**	92.70 ± 0.56
Oven at 38 °C	57.47 ± 3.11	75.65 ± 3.52	87.70 ± 2.17	89.94 ± 0.54	91.15 ± 0.32	92.05 ± 0.97	**92**.**07 ± 0**.**98**	92.07 ± 0.99				
Oven at 50 °C	69.84 ± 1.62	89.91 ± 0.94	92.65 ± 1.33	**92**.**69 ± 1**.**31**	92.69 ± 1.31							
Oven at 60 °C	71.44 ± 3.19	92.79 ± 0.62	**93**.**18 ± 0**.**54**	93.18 ± 0.54								
Oven at 70 °C	91.30 ± 0.15	**91**.**43 ± 0**.**02**	91.42 ± 0.03									
Oven at 80 °C	**91**.**58 ± 0**.**37**	91.58 ± 0.38										
Oven at 93 °C	**91**.**53 ± 0**.**87**	91.53 ± 0.87										

*The bold values in the table represent the final, constant water loss ratio for each drying method.

**Table 3:** The percentage of weight reduction due to water loss during drying of *Trametes versicolor* fruiting bodies by different drying methods and at different drying times* (mean ± SD, n = 3).

Drying method	Drying time (h)										
	3	6	10	14	18	22	26	30	34	38	42
Silica gel at 22 °C	15.47 ± 1.94	16.87 ± 2.24	17.43 ± 2.32	17.88 ± 2.32	18.25 ± 2.36	18.54 ± 2.37	**18**.**69 ± 2**.**44**	18.65 ± 2.45			
Oven at 22 °C	8.22 ± 0.96	9.42 ± 0.86	10.32 ± 0.89	10.87 ± 0.91	11.21 ± 0.90	11.39 ± 0.92	11.52 ± 0.92	11.65 ± 0.94	11.73 ± 0.91	**11**.**92 ± 0**.**95**	11.90 ± 0.96
Oven at 38 °C	12.27 ± 0.68	12.69 ± 0.69	13.23 ± 0.66	13.43 ± 0.69	13.55 ± 0.66	13.64 ± 0.69	13.81 ± 0.69	**13**.**94 ± 0**.**72**	13.92 ± 0.70		
Oven at 50 °C	15.50 ± 1.29	16.35 ± 1.27	16.86 ± 1.27	16.97 ± 1.26	17.10 ± 1.28	**17**.**26 ± 1**.**26**	17.26 ± 1.26				
Oven at 60 °C	15.61 ± 0.26	16.74 ± 0.19	**17**.**23 ± 0**.**16**	17.20 ± 0.20							
Oven at 70 °C	**15**.**60 ± 2**.**30**	15.58 ± 2.29									
Oven at 80 °C	**16**.**63 ± 0**.**37**	16.63 ± 0.37									
Oven at 93 °C	**25**.**01 ± 1**.**14**	25.01 ± 1.14									

*The bold values in the table are the final, constant water loss ratio for each drying method.

At present, the exact reason for the large variability in water loss ratio in *T*. *versicolor* specimens is not known. Two factors are likely to have contributed to the large variability. The first was the difference among the drying methods/temperatures: at low temperatures, not all the water within the mushroom tissue could be converted to vapor, thus causing lower water loss ratios at the 22 °C and 38 °C than at higher temperatures. The second was the potentially different water content among the samples of mushroom tissue. Though we tried our best to pick *T*. *versicolor* fruiting bodies of similar age, size, and location on the log, subtle differences in these characters could have contributed to the water content differences among the fruiting bodies. Under the same conditions, a sample with a lower initial water content would have less water to lose during drying than one with a higher water content and thus would have a lower water loss ratio. Other factors, including mushroom structure and age of the sample, could also have influenced the water loss ratio. For example, a younger and metabolically more active sample might contain more water than an older and metabolically inactive sample. Our data on *A*. *bisporus* seemed to suggest that the second factor likely contributed to the large variability in water loss ratio in *T*. *versicolor*. Specifically, for *A*. *bisporus*, the fruiting bodies were harvested at the same developmental stage and stored under the same condition, and their water loss ratios were within a very narrow range (from 91.43% to 93.18%), with no statistically significant difference among the methods.

Interestingly, silica gel drying resulted in a very high water loss ratio for both species, second only to oven drying at 93 °C and higher than all other tested temperatures. As expected, for both species, with the increase of drying temperature, the drying time was significantly shortened. At temperatures of 70 °C and over, all mushroom samples reached a stable weight within 4 hours of drying (Table 4). Overall, our results indicate both similarities and differences between fleshy and leathery mushrooms in their responses to drying with different methods.

**Table 4:** Number of hours of drying required to reach a stable sample weight; the percentage of nucleic acids that are double-stranded DNA (dsDNA %); and the quantity of dsDNA (ng/mg of dried mushroom tissues) extracted from mushrooms dried using different methods.

Drying method	*Agaricus bisporus*					*Trametes versicolor*				
	t^a^	PNA0^b^	PNA1^c^	QNA0^d^	QNA1^e^	t	PNA 0	PNA1	QNA0	QNA1
Silica gel at 22 °C	37	6.55 ± 2.12^a^	6.35 ± 1.92^a^	492.11 ± 107.65^a^	275.58 ± 15.13^a^*	24	10.46 ± 1.90^a^	8.75 ± 1.43^a^	386.19 ± 103.67^a^	747.63 ± 194.90^a^*
Oven at 22 °C	40	7.23 ± 0.39^a^	5.99 ± 1.19^a^	329.71 ± 41.90^b^	261.83 ± 37.92^a^*	36	7.71 ± 0.46^a^	8.89 ± 0.57^a^	309.63 ± 25.04^a^	658.49 ± 108.69^ab^*
Oven at 38 °C	23	7.14 ± 2.00^a^	6.59 ± 1.69^a^	382.06 ± 108.91^ab^	239.32 ± 18.32^a^*	28	9.17 ± 3.15^a^	10.01 ± 2.22^a^	332.92 ± 37.10^a^	699.93 ± 227.54^ab^*
Oven at 50 °C	11	7.29 ± 0.55^a^	6.67 ± 0.89^a^	390.98 ± 41.89^ab^	271.98 ± 26.49^a^*	19	9.98 ± 1.38^a^	7.52 ± 2.22^a^	333.37 ± 83.55^a^	571.49 ± 96.83^ab^*
Oven at 60 °C	8	6.13 ± 0.60^a^	4.29 ± 0.63^a^	401.72 ± 88.08^ab^	223.01 ± 41.24^a^*	7	8.93 ± 0.67^a^	8.69 ± 1.31^a^	331.03 ± 18.30^a^	619.67 ± 267.43^ab^*
Oven at 70 °C	4	5.48 ± 2.18^a^	6.06 ± 1.30^a^	380.57 ± 87.86^ab^	232.39 ± 65.87^a^*	3	9.85 ± 1.63^a^	9.82 ± 2.01^a^	306.55 ± 72.97^a^	548.97 ± 284.17^ab^*
Oven at 80 °C	3	6.83 ± 1.24^a^	3.93 ± 1.05^a^	276.14 ± 40.55^b^	124.43 ± 30.05^b^*	3	9.76 ± 1.91^a^	9.11 ± 1.60^a^	270.04 ± 27.33^a^	440.56 ± 197.23^ab^*
Oven at 93 °C	3	5.97 ± 0.58^a^	5.80 ± 2.52^a^	132.05 ± 29.65^c^	43.71 ± 3.07^c^*	2	7.46 ± 1.08^a^	8.74 ± 1.48^a^	287.97 ± 47.72^a^	316.50 ± 132.10^b^*

The means in each column followed by the same superscripts are not significantly different and the “*” marks in each row within the same specimen indicate significant differences between the two time points of DNA extractions at P < 0.05 according to Duncan’s multiple range tests.

^a^t, Number of hours of drying by eight treatments to reach a stable sample weight.

^b^PNA0, percentage of nucleic acids that are double-stranded DNA (dsDNA %) extracted immediately after drying.

^c^PNA1, percentage of nucleic acids that are double-stranded DNA (dsDNA %) extracted after one-month storage after drying.

^d^QNA0, quantity of dsDNA (ng/mg of dried mushroom tissues) extracted immediately after drying.

^e^QNA1, quantity of dsDNA (ng/mg of dried mushroom tissues) extracted after one-month storage after drying.

### DNA Quality and Quantity from Dried Mushroom Samples

**Figure 2:**
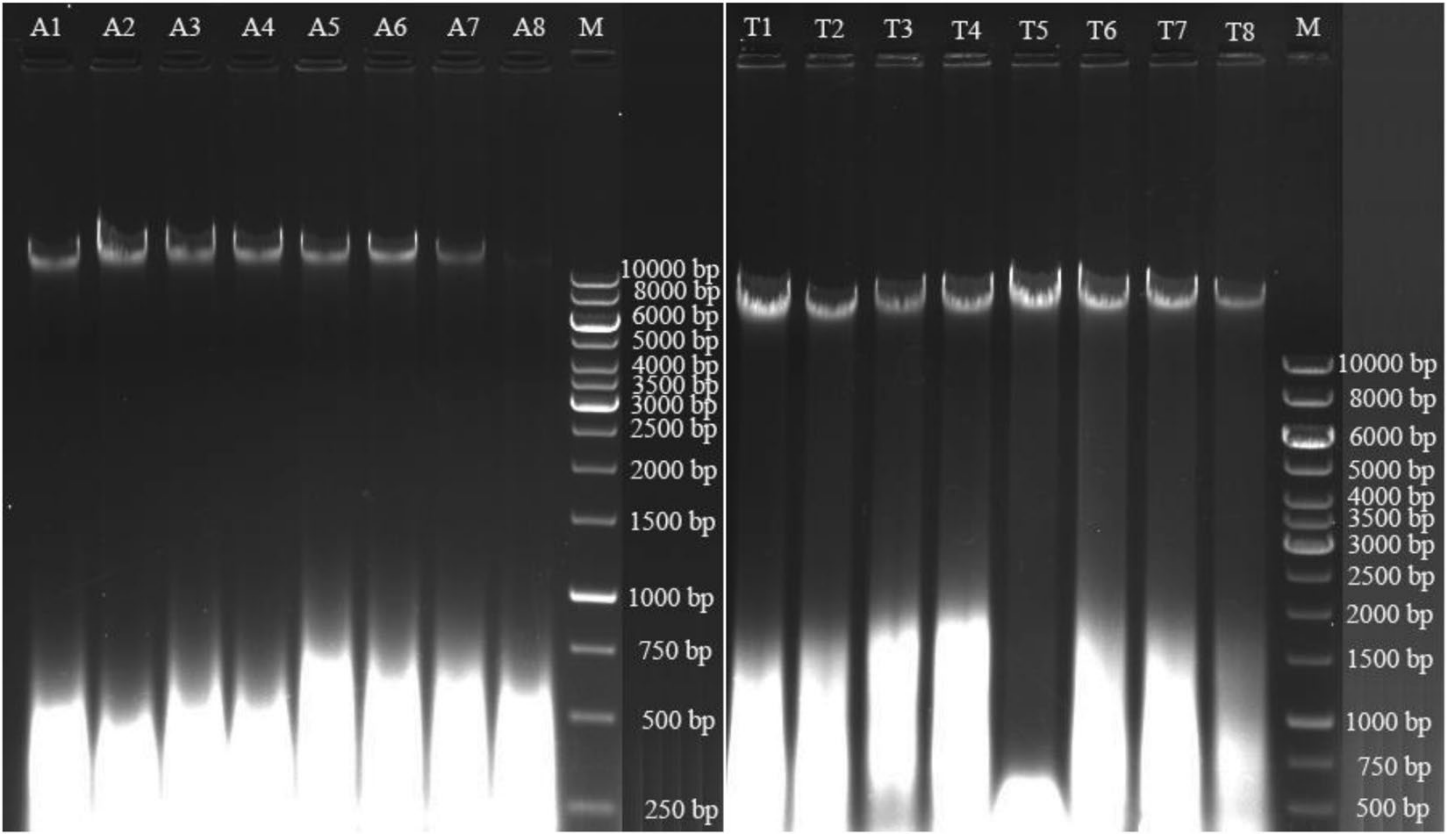
A representative gel of the electrophoretic pattern of whole cell nucleic acids extracted immediately after drying from mushroom specimens dried using different methods. A, *Agaricus bisporus*, A1: Silica gel at 22 °C; A2: Oven at 22 °C; A3: Oven at 38 °C; A4: Oven at 50 °C; A5: Oven at 60 °C; A6: Oven at 70 °C; A7: Oven at 80 °C; A8: Oven at 93 °C; M: GeneRuler 1 kb DNA ladder; T, *Trametes versicolor*, T1: Silica gel at 22 °C; T2: Oven at 22 °C; T3: Oven at 38 °C; T4: Oven at 50 °C; T5: Oven at 60 °C; T6: Oven at 70 °C; T7: Oven at 80 °C; T8: Oven at 93 °C; M: GeneRuler 1 kb DNA ladder.

The extracted nucleic acids from dried mushroom samples were assessed for both their quality and quantity. Through agarose gel electrophoresis analyses, we observed that all samples had a major DNA band with molecular weights much greater than the largest DNA fragment (10 kb) in the ladder (Fig. 2). Because of the high molecular weight of our DNA extracts, we were unable to use BioAnalyzer (which has an upper limit of ~10–12 kb) to check the DNA quality from each of the drying methods based on the relative proportions of individual DNA fragment lengths. Here, instead of using the relative proportions of high molecular weight dsDNA in each sample as the indicator of DNA quality, we used the percentage of total dsDNA in each nucleic acid extract as a surrogate measure of DNA quality. Our results showed that the percentages of dsDNA ranged from 3–12% of the total extracted nucleic acids (Table 4). However, there was little statistical significance nor consistency in the differences among the mushroom drying methods with regard to DNA quality (Table 4). One contributor to this lack of consistency was the relatively large variation in DNA quality among repeats within individual drying methods (Table 4). The only consistent difference was between the two species where DNA quality was higher for *T*. *versicolor* samples than for *A*. *bisporus* samples for all eight drying methods at both time points (Table 4). In all samples of both species, the majority of nucleic acids were RNA (see bottom part of Fig. 2). At present, the reason for the difference between the two species is not known. One possibility is that the fleshy mushroom *A*. *bisporus* contains more water and might have been more metabolically active, thus having more RNA within each cell than the leathery mushroom *T*. *versicolor*.

Compared to the lack of difference in the percentages of dsDNA among the drying methods, we observed several consistent variations with regard to dsDNA quantity among the tested drying methods. For *A*. *bisporus*, silica gel drying at 22 °C produced the highest yield of dsDNA (Table 4). However, aside from the two high temperature treatments (80 °C and 93 °C oven drying) that showed significantly less dsDNA, there was no or little statistically significant difference among *A*. *bisporus* mushrooms dried by the remaining six methods (Table 4). For *T*. *versicolor*, the highest dsDNA yield per gram of dried tissue was also from silica gel drying at room temperature. However, the only significant difference was observed between drying at 93 °C and silica gel drying. The dsDNA yields among the remaining comparisons were statistically not different from each other (Table 4). At high temperatures such as 93 °C oven drying, DNA denaturation most likely occurred during drying, possibly contributing to the lower amount of dsDNA. However, the percentages of dsDNA were very similar among the treatments (Table 4). Thus, it is likely that the denatured dsDNA from the high temperature treatments re-annealed in the DNA storage buffer after extraction was finished.

Our DNA extraction results are in general agreement with those of Harris^13^ who reported that there was little difference between plant samples dried at 70 °C and those dried by silica gel. Similarly, Hosaka and Uno^5^ reported little difference in the yields of genomic DNA for mushroom samples dried at 35 °C, 52 °C, and 71 °C. However, the above studies tested a narrower range of temperature conditions than our study here. Furthermore, our results disagreed with those of Liston and Rieseberg^12^ who reported that spinach leaves dried at 42 °C were significantly better than those dried by silica gel. Importantly, though the yields were lower for both species at 93 °C, we were able to extract high quality dsDNA for mushrooms dried at this temperature for both species, a result different from that of Haines and Cooper^15^. It should be mentioned, however, that they only examined *A*. *bisporus* fruiting bodies.

### Comparison between Nucleic Acids Extracted Immediately after Drying and One Month after Drying

Following mushroom forays in forests and meadows, most mushroom samples are not subjected to DNA extraction as soon as they are dried. Often there is a time lag, with DNA extractions being conducted after returning to the lab from the field. Here, we tested the effect of one month’s storage at room temperature on the quality and quantity of dsDNA from mushroom samples dried by different methods. Our comparisons showed that for dried *A*. *bisporus* samples, the quantity of dsDNA decreased significantly after one month of storage for all samples compared with extraction immediately after drying regardless of the drying method (*F* = 68.05, *df* = 1, *P* < 0.01) (Table 4). However, the dsDNA percentages changed relatively little in *A*. *bisporus* extractions after the one-month storage for all samples dried by the eight methods (Table 4). The data indicate that after drying by these methods, the total amount of nucleic acids, including dsDNAs, in this species decreased continuously. Interestingly, unlike *A*. *bisporus*, the total dsDNA yields increased for *T*. *versicolor* samples extracted after a one-month storage compared with immediate extraction for all eight tested drying methods (*F* = *36*.*07*, *df* = *1*, *P* < *0*.*01*). Furthermore, the increased yields for *T*. *versicolor* after storage were associated with no significant change in the percentages of dsDNA in each sample between the two time points (Table 4).

At present, the reasons for the differences between the two species are unknown. One potential factor might be due to the differences in water content in the original fruiting bodies (Tables 2 and 3). The high water content in the original *A*. *bisporus* fruiting bodies might make the dried specimens prone to absorb moisture from the air, causing their dsDNA as well as other nucleic acids to be degraded through hydrolysis. In contrast, the low moisture-absorption ability and the additional one-month storage after desiccation for *T*. *versicolor* might have made its cell walls easier to break during DNA extraction, permitting more nucleic acids to be extracted from these samples.

### PCR Amplification of ITS and a Single Copy Nuclear Gene

**Figure 3:**
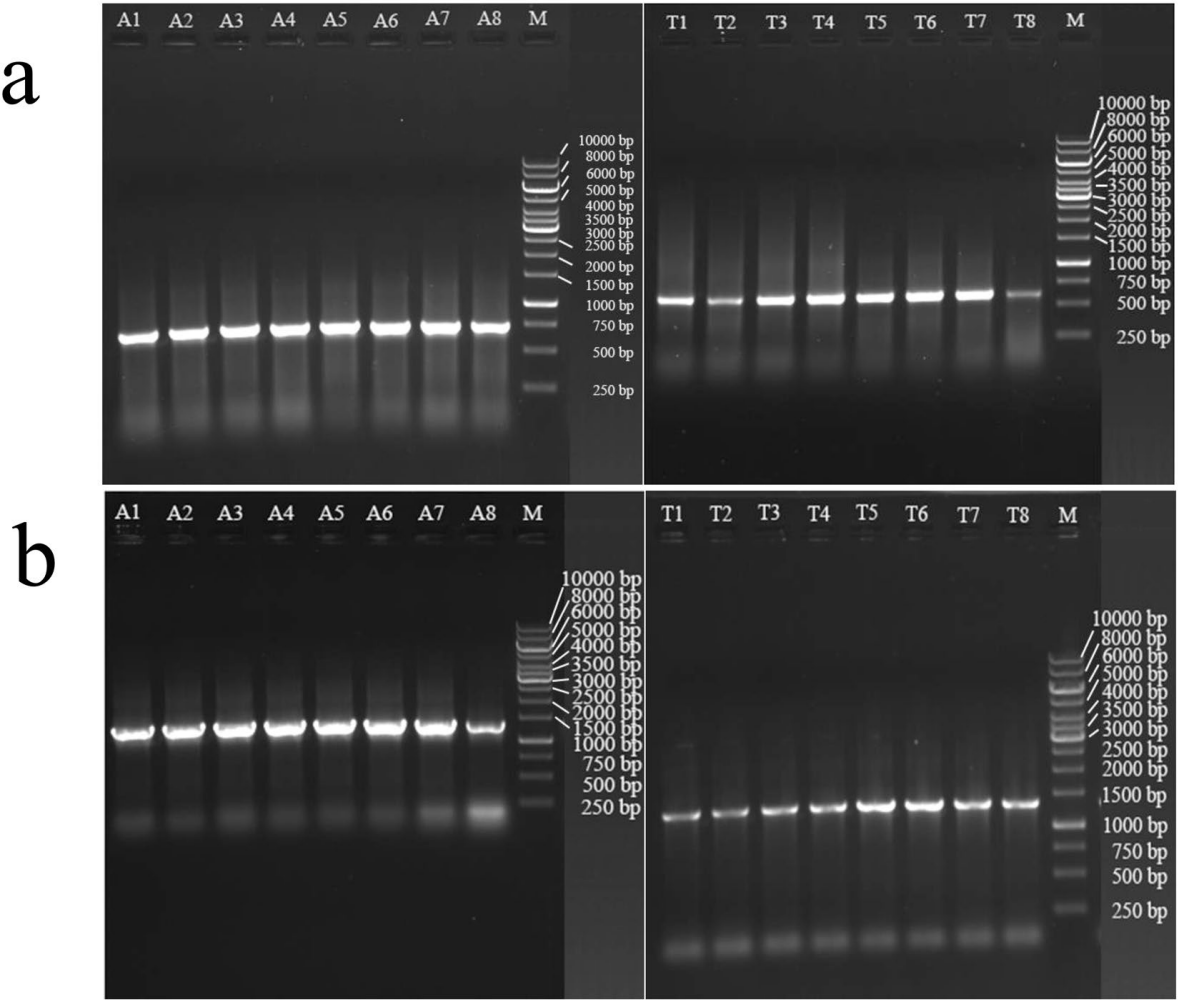
A representative gel of PCR products of ITS (**a**) and a single-copy gene (**b**) from mushroom specimens dried using different methods and stored for one month. A, *Agaricus bisporus*, A1: Silica gel at 22 °C; A2: Oven at 22 °C; A3: Oven at 38 °C; A4: Oven at 50 °C; A5: Oven at 60 °C; A6: Oven at 70 °C; A7: Oven at 80 °C; A8: Oven at 93 °C; M: GeneRuler 1 kb DNA ladder; T, *Trametes versicolor*, T1: Silica gel at 22 °C; T2: Oven at 22 °C; T3: Oven at 38 °C; T4: Oven at 50 °C; T5: Oven at 60 °C; T6: Oven at 70 °C; T7: Oven at 80 °C; T8: Oven at 93 °C; M: GeneRuler 1 kb DNA ladder.

Our attempts to amplify marker loci using the combination of existing (ITS5 and ITS4) and newly designed primers (AB-F, AB-R, TV-F, TV-R) (Table 1) were successful for all the dried samples of both mushroom species. A representative figure showing the relative quantities of amplified PCR products from one-month storage samples is presented in Fig. 3. Overall, there was little difference in ITS amplification among the samples dried by the eight different methods. Interestingly, across the eight drying methods, there was greater amplification of ITS for DNA samples from the fleshy mushroom *A*. *bisporus* than that from the shelf mushroom *T*. *versicolor* (Fig. 3). Several reasons could have contributed to the observed differences between the two species. For example, the copy number of ITS in the *A*. *bisporus* genome could be higher than that in *T*. *versicolor*. Secondly, the amplified ITS product in *A*. *bisporus* is 710 bp, about 150 bp longer than that in *T*. *versicolor* (571 bp). The longer length of ITS in *A*. *bisporus* would bind to more ethidium bromide, and contribute to its greater brightness on agarose gels than the ITS of *T*. *versicolor*.

In the single-copy gene analyses, we found an overall similar pattern of PCR amplification as for the ITS locus. Here, the amplified regions were 1395 bp and 1332 bp respectively for *A*. *bisporus* and *T*. *versicolor*, longer than the products of the ITS locus. For *A*. *bisporus*, the lowest PCR product concentration was found for specimens dried at 93 °C, significantly lower than those dried by other methods. However, *T*. *versicolor* samples dried at 93 °C showed a pattern of amplification efficiency similar to those dried at other temperatures for the single copy gene. Interestingly, the lowest single-copy gene amplification efficiency in *T*. *versicolor* was found for specimens oven-dried at 22 °C, suggesting that drying at this temperature likely caused more hydrolysis of dsDNA in *T*. *versicolor*. Further sequencing of the amplified ITS and single-copy gene fragments from both species confirmed their species and gene identities (Data not shown).

### Mushroom Drying Efficiency Based on dsDNA Quantity

Our results showed that all eight methods worked for drying both types of mushroom samples and all dried samples produced a sufficient quality and quantity of dsDNA for PCR amplification and downstream sequencing. Without considering the time requirement, the two high temperature treatments at 80 °C and 93 °C generated dried mushrooms that produced significantly less dsDNA than other methods for the fleshy mushroom *A*. *bisporus* (Table 4). For *T*. *versicolor*, mushrooms dried in the 93 °C oven had significantly less dsDNA than the other seven methods (Table 4). However, if the length of drying time was considered as a denominator in our calculations, the drying efficiency steadily increased from 22 °C to 70 °C of oven drying, after which it began to decrease (Table 5). One exception was for *T*. *versicolor* dried at 93 °C which had a higher efficacy than the 70 °C treatment at time 0 (immediately after drying). Similar to the significant differences observed in the amounts of dsDNA between samples extracted at time 0 and one month later, significant differences were also observed between EAT at time 0 and after the one-month storage period among the tested drying methods (*F* = 60.32, *df* = 1, *P* < 0.01) for *A*. *bisporus* and (*F* = 8.65, *df* = 1, *P* < 0.01) for *T*. *versicolor*, as expected. Overall, the 70 °C oven-drying method seemed to be the best, producing the highest dsDNA yield per unit of drying time. The advantages of this drying method can also be shown in the relative change of dsDNA between the two DNA extraction time points. For *A*. *bisporus* samples, the loss of dsDNA after one month of storage per unit of drying time was significantly less at 70 °C than at 80 °C and 93 °C but similar among 50 °C, 60 °C and 70 °C (Table 5). For *T*. *versicolor*, the highest gain of dsDNA after one month of storage per unit of drying time was also at 70 °C; however, the differences in the gains of dsDNA among the drying methods are statistically not significantly different from each other (Table 5).

**Table 5:** Efficacy of drying methods estimated based on the amount of dsDNA extracted divided by the time (in hours) that each method took to dry the mushrooms.

Drying method	*Agaricus bisporus*				*Trametes versicolor*			
	RCDQ^a^	EAT0^b^	EAT1^c^	RCDQ/t^d^	RCDQ	EAT0	EAT1	RCDQ/t
Silica gel at 22 °C	0.42 ± 0.12^abc^	13.30 ± 2.91^c^	7.45 ± 0.41^e^*	0.01 ± 0.00^c^	−1.14 ± 1.18^a^	16.09 ± 4.32^d^	31.15 ± 8.12^c^*	−0.05 ± 0.05^a^
Oven at 22 °C	0.19 ± 0.21^c^	8.24 ± 1.05^c^	6.55 ± 0.95^e^*	0.00 ± 0.01^c^	−1.14 ± 0.47^a^	8.60 ± 0.70^d^	18.29 ± 3.02^c^*	−0.03 ± 0.01^a^
Oven at 38 °C	0.34 ± 0.18^abc^	16.61 ± 4.74^c^	10.41 ± 0.80^e^*	0.01 ± 0.01^c^	−1.10 ± 0.61^a^	11.89 ± 1.33^d^	25.00 ± 8.13^c^*	−0.04 ± 0.02^a^
Oven at 50 °C	0.30 ± 0.13^bc^	35.54 ± 3.81^b^	24.73 ± 2.41 ^cd^*	0.03 ± 0.01^bc^	−0.79 ± 0.60^a^	17.55 ± 4.40^d^	30.08 ± 5.10^c^*	−0.04 ± 0.03^a^
Oven at 60 °C	0.42 ± 0.17^abc^	50.22 ± 11.01^b^	27.88 ± 5.15^c^*	0.05 ± 0.02^bc^	−0.85 ± 0.71^a^	47.29 ± 2.61^c^	88.52 ± 38.20^bc^*	−0.12 ± 0.10^a^
Oven at 70 °C	0.33 ± 0.37^abc^	95.14 ± 21.97^a^	58.10 ± 16.47^a^*	0.08 ± 0.09^b^	−1.03 ± 1.53^a^	102.18 ± 24.32^b^	182.99 ± 94.72^a^*	−0.34 ± 0.51^a^
Oven at 80 °C	0.55 ± 0.06^ab^	92.05 ± 13.52^a^	41.48 ± 10.02^b^*	0.18 ± 0.02^a^	−0.62 ± 0.71^a^	90.01 ± 9.11^b^	146.85 ± 65.74^ab^*	−0.21 ± 0.24^a^
Oven at 93 °C	0.66 ± 0.05^a^	44.02 ± 9.88^b^	14.57 ± 1.02^de^*	0.22 ± 0.02^a^	−0.10 ± 0.43^a^	143.98 ± 23.86^a^	158.25 ± 66.05^ab^*	−0.05 ± 0.22^a^

The means in each column followed by the same superscripts are not significantly different and the “*” marks in each row within the same specimen indicate significant differences between EAT0 and EAT1 at P < 0.05 according to Duncan’s multiple range tests.

^a^RCDQ, Relative change of dsDNA quantity over one month storage.

^b^EAT0, Efficacy of drying based on dsDNA quantity per unit time of drying, extracted immediately after drying.

^c^EAT1, Efficacy of drying based on dsDNA quantity per unit time of drying, extracted one month after drying.

^d^RCDQ/t, Efficacy of drying on the relative change of dsDNA quantity over one month of storage.

If the length of drying time is not a constraint in the field, several other methods, including overnight drying at 60 °C, could produce quantities of dsDNA similar to those of samples dried at 70 °C. This is especially relevant in field conditions where mushroom samples are usually set up for drying in the late afternoon or early evening and packed up the following morning. The 7–8 hours of overnight drying at 60 °C (while researchers rest) coincides with the time that allowed mushrooms to reach their stable dried weight under these conditions. In remote areas without electricity, silica gel drying for 1 to 1.5 days could also work well.

## Conclusions and Perspectives

In this study, we compared eight commonly used drying methods on two types of mushroom samples. We obtained time-course drying data for each of the methods and used several indicators to assess the quality and quantity of DNA extracted from dried mushroom tissue. Our analyses identified both similarities and differences between the two mushroom species in response to different drying methods. Based on the combined information, we believe oven drying at 70 °C for 3–4 h, 60 °C for 7–8 h, or silica gel drying for 1–1.5 days will provide excellent quality and quantity of materials for subsequent molecular ecology, population genetics, and systematics studies. Though all dried mushroom tissues produced quality DNA for both species, we recommend that DNA extraction should be performed as soon as possible for dried fleshy mushrooms to ensure high quality and quantity of DNA. In contrast, researchers can wait, for at least a month and most likely more, to extract DNA from dried shelf and leathery mushroom tissues.

We should note that the focus of this paper is on preserving mushroom samples for DNA analyses. Effective methods for preserving intact wild mushrooms for herbaria and for downstream morphological as well as other types of analyses might be different from those for DNA work. In such situations, lowering water content in the mushrooms as much as possible while keeping intact their macroscopic and microscopic structures (and in certain cases, chemical compositions) should be the goals. Whether the DNA quality and quantity indicators obtained here for the eight methods can be applied to other types of mushroom specimen features remains to be examined.
